# Prediction of Hole Expansion Rate for V-Nb Bainitic High-Strength Steel

**DOI:** 10.3390/ma18235369

**Published:** 2025-11-28

**Authors:** Chuangwei Wang, Feilong Wang, Yonggang Mao, Liangyun Wang, Jie Yu, Jun Li, Huarong Qi

**Affiliations:** 1 Panzhihua Iron & Steel Research Institute Co., Ltd. Pangang Group. Panzhihua 617000, China; ww1005@163.com (C.W.); wfeilong@126.com (F.W.); wileywong@163.com (L.W.); 2 Liuzhou Wuling Automotive Co., Ltd. Guangxi Automotive Group, Liuzhou 545007, China; mygcool@126.com; 3 Faculty of Materials Science and Engineering, Kunming University of Science and Technology, Kunming 650093, China; yujie@kust.edu.cn (J.Y.); mse-lijun@kust.edu.cn (J.L.)

**Keywords:** hole expansion rate, forming limit, instability criterion, bainitic high-strength steel

## Abstract

The hole expansion process of high-strength steel is influenced by multiple factors, including the deformation path, UTS/YS ratio, uniform elongation, sheet anisotropy, sheet thickness, strain rate, material micro-defects and the work hardening exponent. Based on forming limit curves or instability criteria, the prediction of the hole expansion ratio (HER) often requires extensive initial boundary conditions that complicate the result. In this study, V-Nb bainitic steel was subjected to hot continuous rolling and underwent water quenching with different coiling temperatures, then subsequently followed by thermal simulation and mechanical testing to fit the work hardening exponent (n) and to obtain the necking deformation instability curve. The radial displacement at the hole edge during simulation was predicted with the ratio of ultimate tensile strength to fracture strength. Furthermore, based on the tensile fracture failure criterion, the HER was predicted with the true fracture strain derived from uniaxial tensile tests. Comparison between the simulated results and actual hole expansion tests shows that the crack resistance in the post-uniform stage, strain hardening capacity and deformation compatibility between the microstructure and matrix are critical factors. And the proposed model achieves a prediction accuracy of over 85% for the V-Nb bainitic high-strength steel.

## 1. Introduction

Energy saving and environmental protection are major trends in the automotive industry, necessitating the use of lightweight high-strength steels to reduce body weight without compromising safety. The hole expansion ratio (HER) test, a key parameter for evaluating the edge stretchability of high-strength steels, commands a set of standardized procedures that are often time-consuming and complex. The delayed feedback from these tests hinders timely adjustments in hot-rolling processes, which makes product quality uncontrollable. Fabricio Longhi Bolina [1] employs a combination of finite element modeling analysis and experiments to enhance research efficiency and achieve bidirectional verification of the structural behavior and mechanical equivalence of ordinary and high-strength reinforcing bars at high temperatures. Musab Rabi [2] proposed an automatic prediction model and used eight different machine learning (ML) algorithms to predict the ultimate flexural capacity of stainless steel-reinforced concrete beams. Based on the design standards and the analysis procedures provided in the continuous strength method (CSM), the results of the developed machine learning model were further verified. Through sensitivity analysis, the influence of key input parameters on the ultimate flexural bearing capacity was investigated. The proposed GB model exhibites better performance and prediction accuracy. When comparing the hole expansion performance of five 3 mm hot-rolled high-strength steels with multi-directional uniaxial tensile test results, Pekka Plosila et al. [3] found that anisotropy significantly influences hole expansion performance at similar strength levels, and the hole expansion ratio (HER) cannot be reliably predicted by a single local formability parameter. Similarly, Siwook Park et al. [4] developed a macro–micro dual-scale finite element (FE) method incorporating micro-roughness and local hardness to improve the accuracy of the hole expansion prediction. And Toros Arda Akşen et al. [5] used finite element simulations to model hole expansion tests on TWIP940 and TRIP590 steels, employing the implicit solver in MSC Marc for accurate HER predictions. However, these studies did not consider the effect of soft and hard phases, where the soft phase can enhance fracture resistance by alleviating stress concentration at the hole edge. To address this, Kejun Hu et al. [6] applied digital image correlation (DIC) and acoustic emission (AE) techniques to monitor crack initiation mechanisms in soft and hard phases during tensile testing of holed specimens. Moreover, research on how the localized deformation of the hole during hole expansion varies is more complicated. Woojin Cho et al. [7] and Tao Zhang et al. [8] emphasized the significant influence of surface damage from hole-punching on HER. By studying together the uniaxial, central hole and notched tensile specimens, Tao Zhang et al. [9] considered the most important effect of strain hardening rate on HER with DIC. Siwook Park et al. [10] demonstrated that the HER was affected by local deformation history and microstructural evolution due to grain-level inhomogeneity. Jin Jae Kim et al. [11] simulated hole expansion with the Kim Tuan hardening model, revealing the critical role of localized thinning in HER prediction. Shin-Yeong Lee et al. [12] found that the GTN-shear model accurately predicts fractures in conical hole expansion under nonlinear loading paths but performs poorly in stretch-bending tests. Ruiting Jiang et al. [13] observed that more surface microcracks in HER specimens were correlated with better hole expansion performance.

In terms of the influence of the sheet material on the hole expansion rate, Surajit Kumar Paul et al. [14] proposed an analytical method based on true fracture strain from uniaxial tensile tests to predict HER. Hong-Sang Park et al. [15] highlighted the importance of the yield stress under plane strain conditions and the plastic strain ratio. Mohammad Emami et al. [16] reported the significant influence of sheet thickness on HER. Jae Hyung Kim et al. [17] emphasized the contribution of post-uniform elongation in predicting HER for thin steels. In terms of the influence of microstructure, Ying Zou et al. [18] demonstrated that lowering the coiling temperature refines grains and improves HER. Xiaoying Hou et al. [19] found that vanadium microalloying in ferrite-bainite dual-phase steel enhances strength–ductility balance and achieves a HER of 33%.

All above studies indicate that the HER is influenced by multiple interrelated factors, thus a simple, fast and reliable method to determine the HER presents a significant challenge for high-strength steel manufacturing. In low-carbon bainitic high-strength steels, variations in coiling temperature and the addition of microalloying elements such as Nb and V lead to different isothermal cooling paths, resulting in diverse microstructures (e.g., M/A islands, lath bainite, granular bainite). These variations ultimately affect the final strength, ductility and HER of the steel coils. In this study, a V-Nb micro alloyed bainitic steel was designed and fabricated. Based on tensile properties, a predictive algorithm for HER is proposed. This work aims to provide a rapid HER measurement method to optimize hot deformation processes and guide the microstructural control of isothermal bainite, thereby offering theoretical support for the industrial production of bainitic high-strength steels.

## 2. Materials and Methods

### 2.1. Experiment Process

In this study, V-Nb bainitic steel was subjected to hot continuous rolling in 1150 °C and 930 °C, the finished rolling temperature, and then though-water cooling to 900 °C to obtain different coiling temperature, 438 °C, 452 °C, 474 °C and 489 °C, samples. The composition of V-Nb steel alloy was detected with a Glow Discharge Spectrometer produced by Thermo Fisher Scientific Corporation, USA and listed as Table 1.

After cooling and sampling, the experimental material was processed into cylindrical bone-shaped room-temperature tensile samples via wire electrical discharge machining (WEDM), with a diameter of ф10 mm at both ends, ф6 mm in the middle section, and a total length of 1160 mm. The two ends of the samples were turned to achieve smooth and flat surfaces, then polished with sandpaper to a surface roughness of 2.6 μm, in order to ensure no scale remained on either end. Subsequently, room-temperature tensile tests were performed with an AJX-V universal testing machine (Shimadzu Corporation, Kyoto, Japan) at a tensile rate of 1 × 10^−3^ s^−1^. The coiled samples were cut in half by WEDM, etched with a 3% nitric acid-ethanol solution and the microstructures of the bainitic steel at different deformation temperatures were observed using a transmission electron microscope (TEM) (Thermo Fisher Scientific Company, Hillsboro, OR, USA).

The hole expansion tests were conducted on a Zwick BUP1000 sheet metal forming tester (Zwick Roell Corporation, Ulm, Baden-Württemberg, Germany). The workpiece had a hole diameter of Φ10mm, a thickness of 2.5 mm and an outer diameter of Φ100 mm. The punch had a 60° cone angle, a cylindrical diameter of approximately Φ35 mm and a length of about 100 mm. The upper die featured an inner diameter of Φ60 mm, an inner filet radius of 5 mm and an outer diameter of Φ100 mm. The reason for selecting a 60° punch cone angle and a speed of 0.5 mm/s is in accordance with the provisions of the section on Method of hole expanding test in the National Standard Sheet metal formability and test methods [20]. Changes in these parameters will cause variations in the hole expansion ratio test results: an excessively large cone angle will reduce the contact compressive stress area, thereby decreasing the hole expansion ratio, and an excessively high punch speed will also lower the hole expansion ratio. Therefore, the hole expansion test must be conducted at the specified angle and speed.

### 2.2. Finite Element Simulation

**Figure 1:**
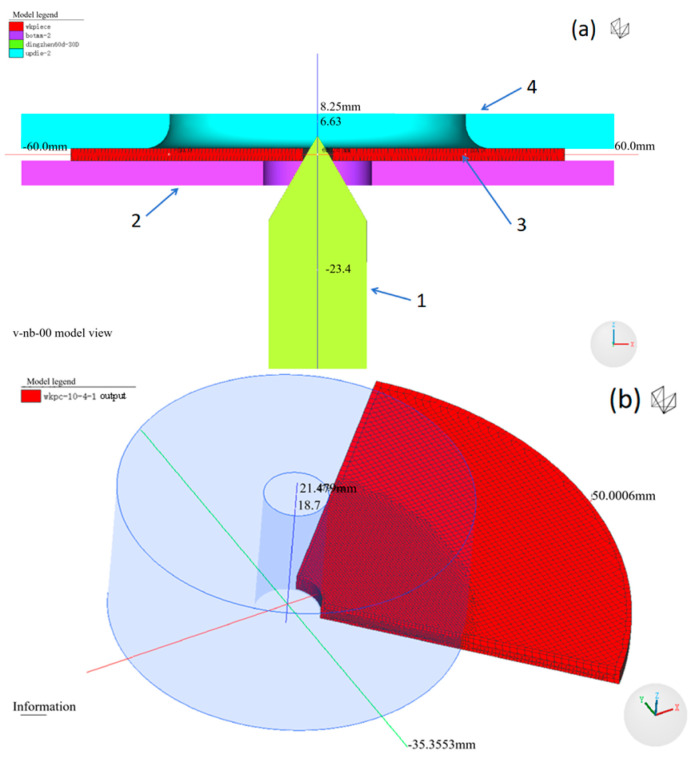
(**a**) Physical model of the hole expansion test: a-1 punch, a-2 lower die, a-3 workpiece, a-4 upper die; (**b**) refined mesh of the workpiece.

The construction of the theoretical model is consistent with the hole expansion test, including mold dimensions, punch diameter and shape, lifting speed of the ejector rod, stroke, etc., as shown in Figure 1. The grid sensitivity and boundary constraints are considered uniformly in the FE calculation process to establish a unified convergence criterion. To improve the calculation speed, the model is set as a 1/4 axisymmetric structure in the simulation, with hexahedral meshes adopted. The element size is 0.7862 mm, and the total number of elements is 19416. The material data are calculated by JMatPro 5.0 (Sente Software Company, Guildford, Surrey, UK), calibrated by actual tensile tests, and imported into the material library of Simufact-forming software for calculation. The temperature is room temperature (20 °C), and the friction coefficient is selected as 0.4 according to the friction coefficient and roughness range specified in the section on Method of hole expanding test in the National Standard sheet metal formability and test methods [20].

## 3. Results and Discussion

### 3.1. Mechanical Properties of V-Nb Bainitic High-Strength Steel at Different Coil Rolling Temperatures

**Figure 2:**
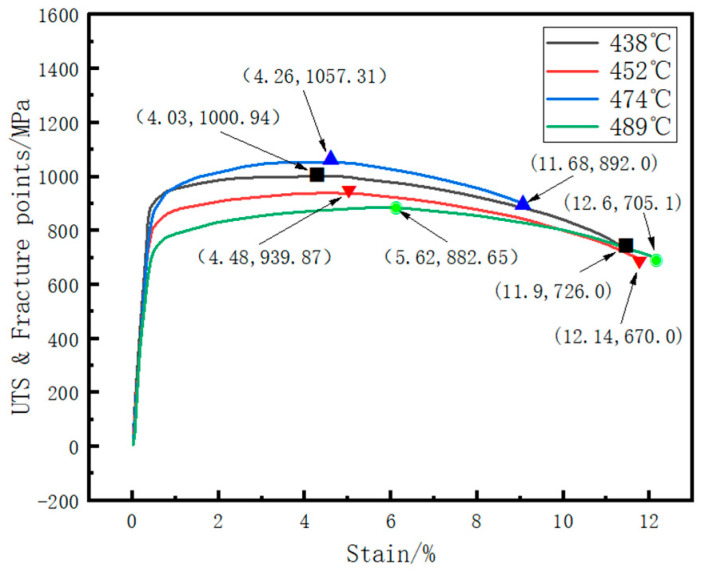
Mechanical property curves of steel coil specimens at different cooling temperatures.

The hot-rolled steel coils were though-water cooled to coiling temperatures of 438 °C, 452 °C, 474 °C and 489 °C, respectively. After cooling to room temperature, samples were taken for tensile testing to obtain the stress–strain curves and hole expansion experiment. The ultimate tensile strength (UTS) and the stress–strain values at complete fracture for each sample are shown in Figure 2.

Based on Figure 2, the uniform and necking deformation stage elongations were separately extracted. The yield strength and yield-to-tensile ratio for each curve were calculated with corresponding hole expansion test results as shown in Table 2.

With the increase in coiling temperature, the hole expansion ratio first increases and then decreases. When the coiling temperature is 452 °C and 474 °C, the hole expansion ratio reaches the maximum of 60%. Meanwhile, the yield strength and tensile strength at 474 °C are the highest. From the actual test data, it can be seen that there is no obvious linear relationship between common physical parameters (such as yield strength, tensile strength, uniform elongation, total elongation, and yield ratio) and the hole expansion ratio. This reflects the complexity of predicting the hole expansion ratio of high-strength steel and increases the difficulty of accurately predicting the material’s hole expansion ratio.

### 3.2. Hole Expansion Test Results

**Figure 3:**
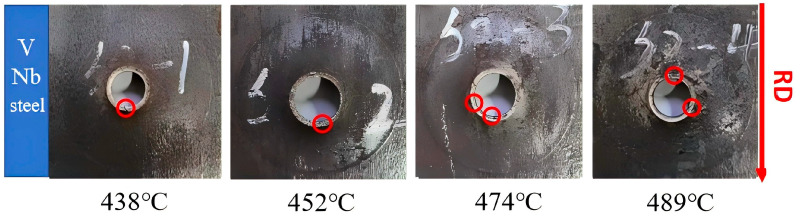
The hole expansion morphology of the V-Nb steel sample with different coiling temperature, RD is the rolling direction.

The appearance of the specimens after hole expansion were shown in Figure 3. In most tested samples, cracking occurred primarily parallel to the rolling direction, as the red circles in Figure 3. For the V-Nb steel, cracking in both the perpendicular and parallel directions reflect the influence of microstructure evolution—associated with the rolling direction—on crack propagation. As shown in Table 3, the HER of the V-Nb steel first increases and then decreases with a rise in the isothermal transformation temperature.

During the rolling of steel coils, the elongation along the Rolling Direction (RD) is particularly large, while that along the Transverse Direction (TD) is relatively small. This causes the grain deformation planes to align along the RD, resulting in the formation of rolling texture. As a result, the material exhibits strong deformation capacity in the RD but relatively weak capacity in the TD. During the hole expansion process, the points parallel to the RD are subjected to the maximum tangential tension in the TD. However, because of the rolling texture, these points are most prone to cracking—that is why the cracking points are parallel to the RD. Meanwhile, due to the structural symmetry of cubic crystals, when most close-packed planes are parallel to the rolling direction (RD), some close-packed planes are also aligned along the TD, though these planes are not as neatly arranged as those along the RD. Therefore, in a few cases, fracture points may also appear perpendicular to the rolling direction.

### 3.3. Numerically Predicted Hole Expansion Ratio

**Figure 4:**
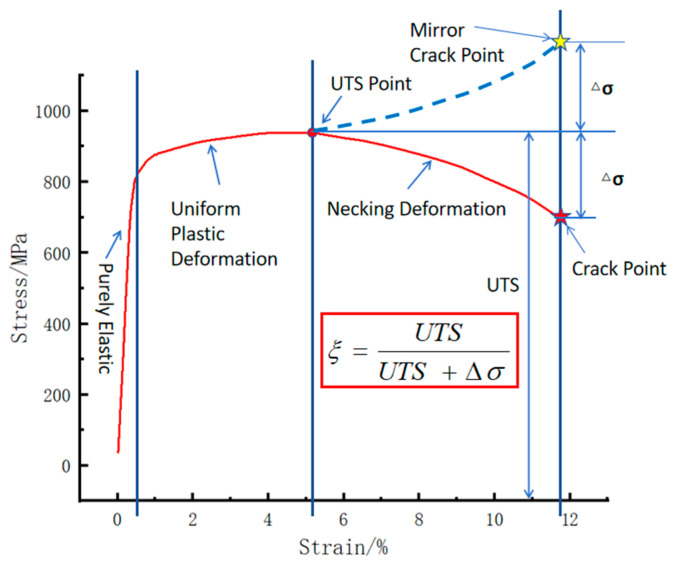
Proportionality coefficient ξ determined from the mirrored stress drop in the post-uniform stage.

During the process of hole expansion, the material undergoes two distinct stages: uniform plastic deformation and necking deformation. The final hole expansion ratio (HER) is determined collectively by the capacity of material for uniform deformation and its subsequent necking deformation. In the necking deformation stage, crack initiation and propagation occur simultaneously, ultimately leading to material fracture. Fracture represents the macroscopic separation of the material, which follows a process of crack initiation and subsequent propagation. In other words, while the stress conditions for crack initiation are similar in tensile tests and hole expansion tests, the subsequent stress environment for crack propagation differs significantly—compressive stress in hole expansion versus tensile stress in tensile testing. This difference makes it difficult to accurately predict the HER theoretically just with tensile test data alone [18]. A longer plastic hardening stage on the stress–strain curve, as shown in Figure 4, illustrates a better material plasticity. Specifically, under the same uniform strain condition, a higher strain hardening exponent (n) leads to more uniform deformation and fewer residual crack initiation sites in the deformed zone [21]. Conversely, a slower stress drop in the necking deformation stage indicates better material toughness and crack resistance. A rapid stress drop in this stage suggests accelerated crack initiation and propagation, leading to premature fracture and poor elongation.

During the plastic deformation of materials, the stress–strain curve exhibits distinctly different response characteristics in the uniform deformation stage and unstable fracture stage. These two stages are marked by the maximum tensile strength value as the turning point on the stress–strain curve. This phenomenon is particularly evident during the reaming process.

After instability occurs in the tensile test, the curve from the ultimate tensile strength point to the fracture point reflects the overall ability of material to resist crack initiation (dispersed instability) and crack propagation (concentrated instability). During hole expansion, only the metal at the upper edge of the small hole inner wall is in a circumferential tensile stress deformation state, and its stretching and failure process is consistent with that of the traditional tensile test; the metal far from the small hole remains in the uniform deformation stage. It can thus be inferred that the inflection of the upward jacking force is caused by the metal at the upper edge of the small hole entering the instability stage. However, since the remaining metal is in uniform deformation, the load inflects but continues to rise. In order to maintain the “upward” trend of the traditional stress–strain curve, the curve between the ultimate tensile strength value and the fracture strength value was “mirrored” to make it consistent with the ascending trend of the expansion hole top-up force. Based on the proportional relationship between the ultimate tensile strength and fracture strength, the position of the expansion hole cracking can be determined.

In Figure 4, the inflection point between the uniform plastic deformation stage and the necking deformation stage, i.e., the ultimate tensile strength (UTS) point, was first identified on the experimentally obtained stress–strain curve. The fracture strength value was then determined, and the difference (Δσ) between UTS and fracture strength was calculated. The dimensionless coefficient ξ = Δσ/(UTS + Δσ) was used to represent the radio of the stress drop stage compared to the total deformation stage. This coefficient reflects the ability of material to resist fracture through localized crack initiation and micro-crack propagation under increasing stress.

The main theoretical basis for the introduction of the dimensionless coefficient ξ is the principle of conservation of deformation energy: a certain volume of material first undergoes elastic deformation after absorbing a certain amount of energy; if it absorbs a certain amount of energy again, plastic deformation will occur; if it continues to absorb a certain amount of energy, unstable fracture will take place. From the energy perspective, for the same material, the magnitudes of these three parts of energy are related to the volume of the material. In particular, the stress–strain change in the unstable fracture part is directly related to the ability of material to resist crack initiation and crack propagation, and the law of the correlated change in stress and strain is similar in the same deformation stage. After the material enters the unstable fracture stage, if the absorbed energy is the same, the longer the curve from instability to fracture (i.e., the lower the fracture point at the same deformation rate), the better the fracture resistance of the material, and the corresponding value of the hole expansion ratio of the material will be larger. However, during hole expansion, only the metal around the small hole that participates in the fracture has the same deformation history as the metal in the tensile test, while the rest is only in the uniform deformation stage. Therefore, only the dimensionless coefficient ξ is used, and the energy ratio relationship between the unstable fracture zone and (uniform deformation zone + unstable deformation zone) is adopted to predict the fracture position of the material around the small hole during hole expansion. The value of the hole expansion ratio is then predicted based on the radial displacement at that moment.

The punch load curve obtained from the hole expansion simulation was analyzed. The first inflection point on the curve, designated as *F*_UTS2_, marks the transition from uniform deformation to necking deformation in the workpiece. *F*_Predic_ represents the predicted load (KN). The fracture load was then calculated by dividing this inflection point load by the previously defined coefficient ξ.

(1) $$F_{Predic}=\frac{F_{UTS2}}{\xi }$$

The radial displacement (R_2_) at the moment of this fracture load *F_P_*_redic_ is corresponding to the simulated hole expansion ratio, given by R_2_/R_0_. *R*_2_ is the radius (mm) of the sample hole after reaming; R_0_ is the radius (mm) of the sample hole before reaming.

**Figure 5:**
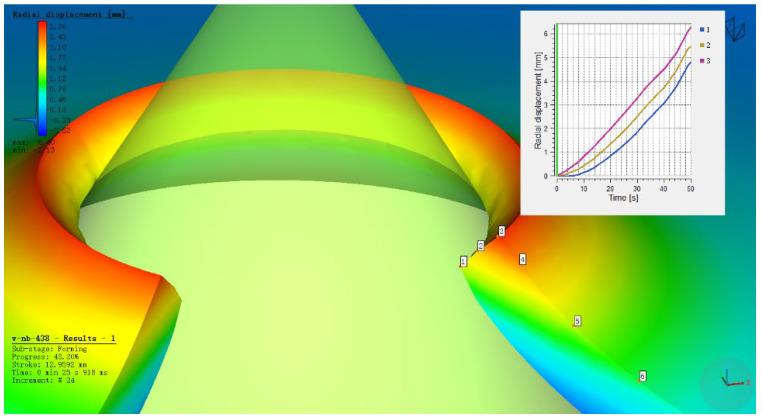
Radial displacement at points 1, 2 and 3 on the workpiece during hole expansion.

The HER (denoted as *H*_0_) is determined by averaging the maximum and minimum diameters of the expanded hole after cracking in actual hole expansion tests. In the simulation, as shown in Figure 5, the averaged radial displacements at points 1, 2 and 3 were used to determine the expanded hole diameter. It was found that the averaged value matched the radial displacement at point 2. Therefore, the diameter at point 2 under the predicted fracture load (*F*_predic_) was used to estimate the HER (*H*_0_).

(2) $$H_{0}=\frac{R_{2}}{R_{0}}\times 100\%$$

**Figure 6:**
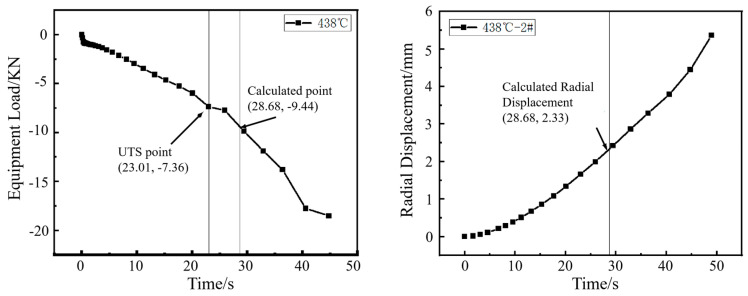
Load–time curve from the hole expansion test and simulated radial displacement for the specimen coiled at 438 °C.

**Figure 7:**
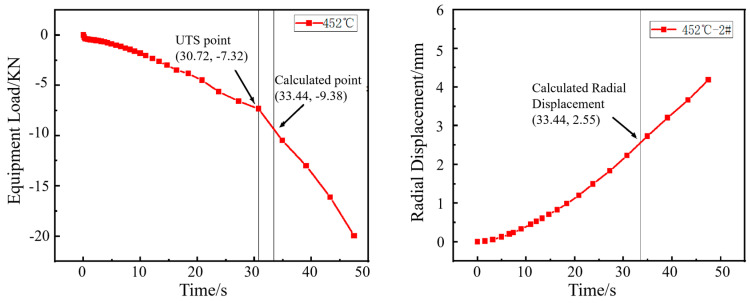
Load–time curve from the hole expansion test and simulated radial displacement for the specimen coiled at 452 °C.

**Figure 8:**
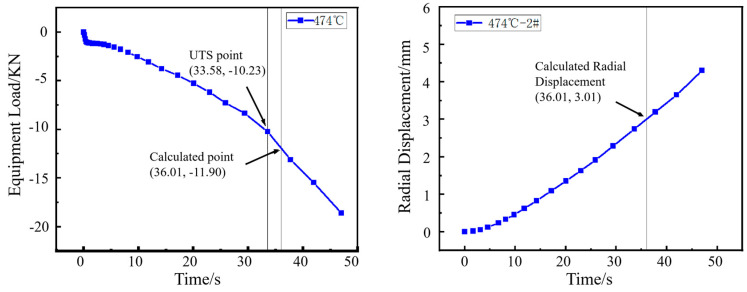
Load–time curve from the hole expansion test and simulated radial displacement for the specimen coiled at 474 °C.

**Figure 9:**
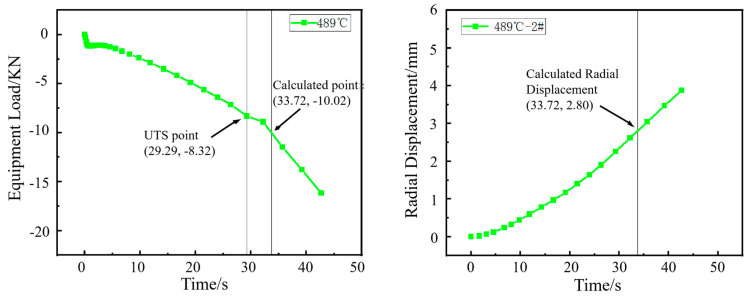
Load–time curve from the hole expansion test and simulated radial displacement for the specimen coiled at 489 °C.

The inflection point in the load curve signifies the transition during hole expansion where the workpiece initially deforms with a stable hardening exponent and begins to experience necking deformation around the hole edge. The formation of crack initiation sites in the necking deformation zone absorbs deformation energy, resulting in the observed inflection point. As shown in Figure 6, Figure 7, Figure 8 and Figure 9, for the same material, this inflection point (UTS) differs from the UTS in the tensile stress–strain curve (Figure 2). That is, the deformation scope after the UTS point in a tensile test, all points in the specimen’s necking deformation zone, contribute to the instability, leading to a global stress drop [11]. In contrast, during hole expansion, only a small region near the hole edge undergoes necking deformation, while the majority of the material outside the hole remains in the uniform deformation stage as shown in Figure 5. (Point 5, 6). This localized necking deformation prevents a global load drop and instead manifests as an inflection point in the load curve which is a signal of local instability [11,16]. From instability to fracture, the stress drop behavior during the post-uniform stage is consistent. Therefore, the theoretical calculations and predicted HER values are summarized in Table 4.

The inability of conventional experimental methods to accurately predict the hole expansion ratio (HER) from tensile test data stems from a fundamental difference in deformation mechanisms. In tension testing, once the ultimate tensile strength (UTS) is reached, the entire instability zone undergoes necking deformation until fracture occurs. In contrast, during hole expansion, only the material immediately surrounding the hole enters the necking deformation stage and eventually fractures, terminating the test. The vast majority of the material outside the hole remains in the strain-hardening-dominated uniform deformation stage. As a result, the load curve does not drop but exhibits an inflection point, after which the load continues to increase. Thus, by obtaining the stress–strain curve of the V-Nb bainitic test steel through room-temperature tensile testing, the HER can be accurately predicted; as shown in Table 4, the accuracy is above 84.41. This approach is straightforward and yields highly accurate results thus should show significant potential for practical engineering applications.

### 3.4. Influence of the Strain Hardening Exponent (n) on Hole Expansion

**Figure 10:**
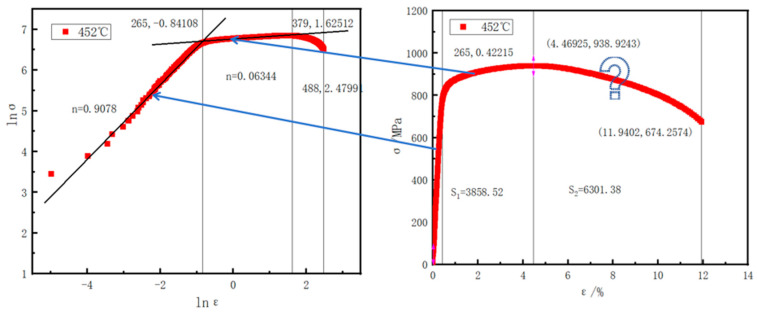
Omission of the post-uniform stage in the determination of the strain hardening exponent (n).

As shown in Figure 10, the lnσ-lnε curves derived from the true-stress–true-strain curves of the V-Nb steel display what the nonlinear relationship between lnσ and lnε is; that is, the strain hardening behavior of the tested steel does not follow a single strain hardening characteristic. The lnσ-lnε curves were divided into two stages with different slopes and fitted piecewise to obtain the parameters of the Hollomon equation (Table 5). According to Figure 10, the temperature dependence of the strain hardening behavior during tensile testing is not pronounced with the increase coiling temperature, which is consistent with the report of [17]. The strain hardening exponent (n1) in the first stage generally shows a slowly decreasing trend with the coiling temperature. In the second stage, the n-value(n2) exhibits a slight increasing trend. The transition strain of the V-Nb steel also decreases slightly as the coiling temperature rises.

As shown in Figure 10, the determination of the strain hardening exponent (n) considers only the uniform hardening stage(n1) and excludes the post-uniform stage(n2). In reality, the hole expansion test continues until the hole edge completely fractures—meaning the post-uniform stage also contributes to the HER. As shown in Table 5, the n2 is 0.1014 for 474 °C which cannot be neglected. Conventional forming limit models based on the hardening exponent only account for the uniform deformation stage. Forming limit criteria can only predict the onset of the post-uniform stage but not the final fracture point, which explains the inaccuracy of n-based predictions. The strain hardening exponent reflects the behavior of the material in the uniform deformation regime and the relationship between stress and strain; the n-value dictates the capacity of the material for uniform plastic deformation. However, due to the inability to account for the nonlinear deformation in the post-uniform stage, methods relying solely on the n-value to predict the HER struggle to deliver accurate results.

### 3.5. Derivation of Forming Limit Curves (FLC) for the V-Nb Test Steel at Different Coiling Temperatures

Existing studies indicate that increasing the strain hardening exponent (n) during the uniform deformation stage enhances the local strain capacity of the sheet, raises the instability limit strain, promotes more uniform strain distribution and improves the overall forming limit. The physical essence of the forming limit lies at the critical point where microscopic damage evolution leads to macroscopic instability. Under external loading, stress–strain effects develop within the material. At this critical point, specific relationships are established among the maximum and intermediate principal stresses, as well as the maximum and intermediate principal strains. These four parameters define the stress-based or strain-based forming limit diagram.

The Swift instability criterion:

(3) $$Swift\left\{\begin{array}{c} Simple\;Tension:\;\frac{{d\sigma }_{1}}{{d\varepsilon }_{1}}=\sigma_{1},\;\bar{\sigma }=B{\bar{\varepsilon }}^{n},\;\varepsilon_{1d}=n\\ Biaxial\;Tension:\;\frac{{d\sigma }_{2}}{\sigma_{2}}={d\varepsilon }_{2},\;\frac{{d\sigma }_{1}}{\sigma_{1}}={d\varepsilon }_{1} \end{array}\right.$$

Under uniaxial tension, the maximum principal strain ε_1_ equals the strain hardening exponent n, leading to diffuse necking deformation.

The Hill instability criterion:

(4) $$Hill\left\{\begin{array}{c} Simple\;Tension:\;\frac{{d\sigma }_{1}}{\sigma_{1}}=-\frac{dt}{t}=-{d\varepsilon }_{3}=\frac{1}{2}{d\varepsilon }_{1}\to \varepsilon_{1l}=2n\\ Biaxial\;Tension:\;\frac{{d\sigma }_{1}}{\sigma_{1}}=\;\frac{{d\sigma }_{2}}{\sigma_{2}}=-\frac{dt}{t}={-d\varepsilon }_{3} \end{array}\right.$$

Under uniaxial tension, ε_1_ equals 2n, resulting in localized necking deformation. σ1, σ2 and σ3 represent the maximum principal stress, the intermediate principal stress and the minimum principal stress, respectively, while ε1, ε2 and ε3 denote the maximum principal strain, the intermediate principal strain and the minimum principal strain, respectively.

(5) $$\frac{d\bar{\sigma }}{d\bar{\varepsilon }}=\frac{n\bar{\sigma }}{\bar{\varepsilon }}=\frac{n\sigma_{1}\sqrt{1-\alpha +\alpha^{2}}}{\bar{\varepsilon }}$$

Assuming the hardening curve of the sheet follows $\bar{\sigma }$=B $\bar{\varepsilon }$ n, the hardening rate during plastic deformation is given by:

(6) $$\varepsilon_{1l}=\frac{\left(2-\alpha \right)}{\left(1+\alpha \right)}\cdot n=\frac{1}{\left(1+\beta \right)}\cdot n$$

(7) $$\varepsilon_{2l}=\frac{\left(2\alpha -1\right)}{\left(1+\alpha \right)}\cdot n=\frac{\beta }{\left(1+\beta \right)}\cdot n$$

(8) $$\varepsilon_{3l}=-\left(\varepsilon_{1l}+\varepsilon_{2l}\right)$$

For the range 0 ≤ α ≤ 0.5 (−0.5 ≤ β ≤ 0), the theoretical limit strain at instability is calculated using the localized necking deformation condition. In this case:

For the range 0.5 ≤ α ≤ 1 (0 ≤ β ≤ 1), the theoretical limit strain is determined based on the diffuse necking deformation condition. In this case:

Given a specific n-value and an assigned ε_1_, the corresponding ε_2_ can be calculated,

(9) $$\varepsilon_{1d}=\frac{2\left(2-\alpha \right)\left(1-\alpha +\alpha^{2}\right)}{\left(1+\alpha \right)\left(4-7\alpha +4\alpha^{2}\right)}\cdot n=\frac{2\left(\beta^{2}+\beta +1\right)}{\left(1+\beta \right)\left(2\beta^{2}-\beta +2\right)}\cdot n$$

(10) $$\varepsilon_{2d}=\frac{2\left(2\alpha -1\right)\left(1-\alpha +\alpha^{2}\right)}{\left(1+\alpha \right)\left(4-7\alpha +4\alpha^{2}\right)}\cdot n=\frac{2\beta \left(\beta^{2}+\beta +1\right)}{\left(1+\beta \right)\left(2\beta^{2}-\beta +2\right)}\cdot n$$

(11) $$\varepsilon_{3d}=-\left(\varepsilon_{1d}+\varepsilon_{2d}\right)$$

thereby constructing the forming limit diagram.

**Figure 11:**
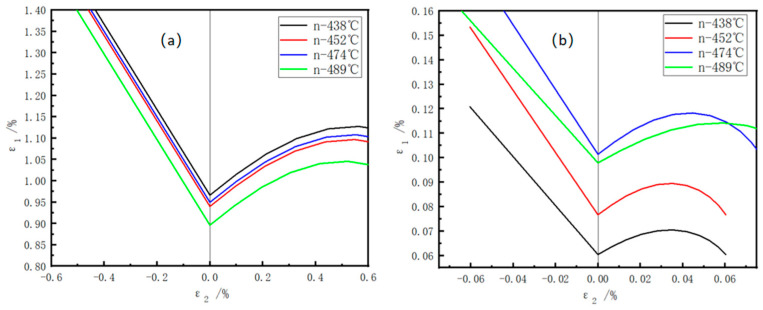
Forming limit diagrams of the V-Nb test steel: (**a**) forming limits during uniform deformation; (**b**) forming limits during necking deformation.

As shown in Figure 11, the specimen coiled at 438 °C exhibits the highest forming limit during the uniform deformation stage (Figure 11a) but the lowest during the necking deformation stage (Figure 11b), resulting in a relatively low final HER. In contrast, the specimen coiled at 452 °C demonstrates high forming limits in both stages, leading to the best hole expansion performance among the four compared samples. The 452 °C specimen shows intermediate forming limits and HER values in both deformation stages.

### 3.6. Influence of Precipitates on the HER

M6C carbides are generally regarded as an unfavorable precipitate phase and can serve as nucleation sites for microcracks. Under impact loading, stress concentration is prone to be generated at the interface between these harder carbides and the relatively softer ferrite matrix, resulting in interfacial debonding or cracking of the carbides themselves. A low-energy propagation path for cracks is thereby provided, which significantly degrades the impact energy and fracture toughness of the material. In contrast, MC carbides are characterized by smaller sizes and a dispersed distribution. These ultrafine carbides can impart potent Orowan strengthening, and owing to their extremely small size and excellent coherency with the matrix, the damage they inflict on toughness is much less detrimental than that caused by M6C carbides.

**Figure 12:**
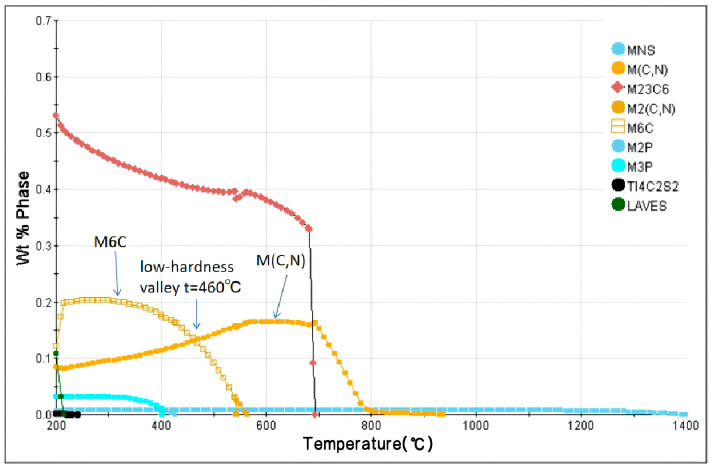
Low-hardness region observed in V-Nb bainitic steel at 460 °C by JMatPro.

According to the calculation results of JMatPro shown in Figure 12, the upper limit of the precipitation temperature of M6C carbides is 568 °C, and their mass fraction increases significantly with decreasing temperature. When the temperature is higher than 568 °C, the precipitated phases are mainly MC carbides, and their mass fraction reaches the precipitation peak around 700 °C. At approximately 460 °C, there exists a precipitation valley point for the high-hardness M6C phase (hardness: 800–1000 HV) and the higher-hardness M(C,N) phase (hardness: 1200 HV), as indicated by the arrow in the figure. The precipitates of the ultra-hard phases are reduced, the hardness coordination between bainite and the strengthening phases is improved and the comprehensive hole expansion ratio is increased.

**Figure 13:**
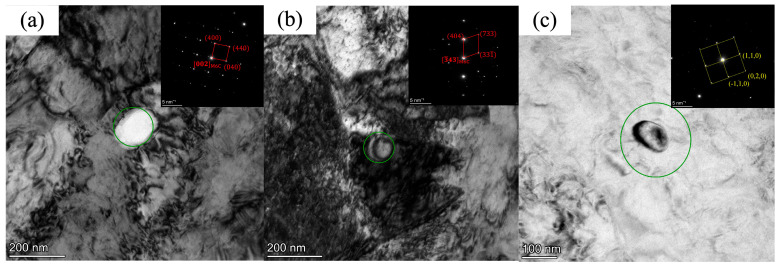
Variation in carbide precipitate size with coiling temperature in V-Nb steel. (**a**) 438 °C; (**b**) 452 °C; (**c**) 474 °C.

For V-Nb steel, when the coiling temperature is 438 °C, it is significantly lower than the dissolution temperature of M6C carbides (the dissolution temperature is 586 °C as shown in Figure 12. At this temperature, M6C carbides possess a high precipitation driving force and initiate nucleation at or near grain boundaries. With the extension of holding time, due to the high diffusion coefficient of grain boundaries, M6C carbides start to coarsen, and grow into large-sized carbides, and dislocation pile-up exists around them, as illustrated in Figure 13a. At 452 °C, the particle size of the M6C phase decreases, while dislocation pile-up remains obviously dense, as shown in Figure 13b. When the actual coiling temperature is increased to 474 °C, precipitation is dominated by MC carbides. Since bainite transformation is mainly governed by shear and diffusion mechanisms, high-density dislocations are introduced during the phase transformation. The increase in dislocation density provides more nucleation sites, that is, MC exhibits a higher nucleation rate compared to M6C. Meanwhile, at the relatively high coiling temperature, with the prolongation of time, large-sized MC carbides grow by merging with smaller MC particles through the short-circuit diffusion of dislocations. However, compared to M6C carbides, since the nucleation rate on dislocations is higher than that on grain boundaries and the dislocation diffusion rate is lower than that of grain boundaries, the MC carbides still have a smaller size even at the higher coiling temperature, and the surrounding dislocations become sparse, as presented in Figure 13c.

## 4. Conclusions

(1) The ultimate tensile strength (UTS) point on the engineering stress–strain curve serves as a critical node separating uniform plastic strain from necking deformation. Beyond the UTS point, the material enters the necking deformation instability stage. The resistance of material to crack propagation without fracturing in this post-uniform stage significantly influences the hole expansion ratio (HER);

(2) The strain hardening exponent (n) can be used to characterizes the ability of material to distribute stress and strain. A higher n-value promotes more uniform deformation, thereby increasing the HER. Additionally, the uniformity of the microstructure of material also plays an important role in determining the HER;

(3) With tensile testing and the hole expansion test with the UTS point, together with numerical simulation, the HER can be effectively predicted. The predicted HER values from the simulations show excellent agreement with experimental results, and the accuracy ranges from 84.41% to 98.64%.

## Figures and Tables

**Table 1:** Composition of V-Nb steel alloy.

Elements	C	Si	Mn	Al	Cr	Mo	Nb	V	Ti
wt%	0.044	0.51	1.79	0.015	0.41	0.254	0.027	0.081	0.001

**Table 2:** Mechanical properties and hole expansion ratio of the tested steel at different coiling temperatures.

Coiling Temperature /°C	Yield Strength /Mpa	Tensile Strength /Mpa	Uniform Elongation /%	Total Elongation /%	Yield Ratio	Hole Expansion Ratio /%
438	916.94	1000.94	4.03	11.90	0.92	47.24
452	835.88	939.87	4.48	12.14	0.88	60.42
474	917.59	1057.31	4.26	11.68	0.87	61.5
489	749.88	882.65	5.62	12.56	0.85	53.77

**Table 3:** Calculated values of the stress drop proportionality coefficient (ξ) for the post-uniform stage.

Coiling Temperature	438 °C	452 °C	474 °C	489 °C
**UTS/MPa**	1000.94	939.87	1057.31	882.65
**Crack Stress/MPa**	726.00	670.00	892.00	705.10
**Δσ**	278.60	266.30	166.10	177.80
**Mirror Stress**	1279.54	1206.17	1223.41	1060.45
**ξ**	0.78	0.78	0.86	0.83

**Table 4:** Comparison of measured and predicted hole expansion ratios (HER) for the V-Nb steel.

Coiling Temperature/°C	Measured HER /%	Measured Diameter/mm	Predicted Diameter /mm	Predicted HER /%	Accuracy Rate/%
438	47.24	14.72	14.66	46.60	98.64
452	60.42	16.04	15.10	51.00	84.41
474	61.50	16.15	16.02	60.20	97.89
489	53.77	15.77	15.60	56.00	96.02

**Table 5:** Work hardening parameters from Hollomon analysis of the V-Nb steel under various coiling temperatures.

Coiling Temperature/°C	StageI;		R-Square	StageII		R-Square	Transition Strain/%
	n1	K1		n2	K2		ε_t_ (StageI–II)
**438**	0.9665	195,829	0.9966	0.0604	1270	0.9795	1.72
**452**	0.9401	144,350	0.9975	0.0767	1253	0.9927	1.70
**474**	0.9497	160,251	0.9952	0.1014	1536	0.9825	1.69
**489**	0.8963	97,945	0.9979	0.0979	1242	0.9943	1.69

## Data Availability

The original contributions presented in this study are included in the article. Further inquiries can be directed to the corresponding author.
